# Development and evaluation of a teleoncology system for breast cancer during the COVID-19 pandemic

**DOI:** 10.2217/fon-2021-0822

**Published:** 2022-02-07

**Authors:** Taleb Khodaveisi, Farahnaz Sadoughi, Kambiz Novin, Mohammad Hosseiniravandi, Afsaneh Dehnad

**Affiliations:** ^1^Department of Health Information Technology, School of Allied Medical Sciences, Hamadan University of Medical Sciences, Hamadan, Iran; ^2^School of Health Management & Information Sciences, Iran University of Medical Sciences, Tehran, Iran; ^3^Department of Radiotherapy and Oncology, Faculty of Medicine, Iran University of Medical Sciences, Tehran, Iran; ^4^Department of Health Information Technology, School of Paramedical Sciences, Torbat Heydariyeh University of Medical Sciences, Torbat Heydariyeh, Iran; ^5^Center for Educational Research in Medical Sciences (CERMS), School of Health Management & Information Sciences, Iran University of Medical Sciences, Tehran, Iran

**Keywords:** breast cancer, oncology, teleoncology, telemedicine, treatment plan

## Abstract

**Background:** The present study describes the steps of developing a hybrid teleoncology system to provide treatment plans for breast cancer patients. **Materials & methods:** This research was conducted in four stages, including developing a proposal for experts, identifying and analyzing system requirements, designing a prototype and implementing and evaluating the final version of the hybrid teleoncology system. **Results:** The results of the usability evaluation showed that the users evaluated the system at a good level and, in practice, the implemented system was perceived to be useful by specialists in providing treatment plans for cancer patients. **Conclusion:** The hybrid teleoncology system is a practical alternative to traditional methods for providing treatment plans to breast cancer patients.

Breast cancer is the most common type of cancer in women and is one of the leading causes of cancer death. It is considered as emotional trauma for women, affecting their lives on various levels, both physically and mentally [1,2]. In general, the incidence of breast cancer is increasing worldwide and Iran is no exception [3]. Its incidence is expected to triple by 2030, and the results of some studies have shown that the average age of patients with this type of cancer in Iran is about 10 years lower than the average age of patients in developed countries [4,5]. In Iran, 6160 patients are diagnosed with breast cancer annually and 1063 of them die from the disease [6]. In addition, cancer care in Iran faces several challenges, including the unequal distribution of resources and specialists across the country, care and treatment costs and inequality in healthcare delivery services [7]. With the outbreak of COVID-19 since December 2019, healthcare systems around the world are facing serious challenges and a large portion of their resources is allocated to managing this crisis. Therefore, the provision of a healthy and safe environment for vulnerable patients in order to deliver care services with appropriate treatment plans is a major concern [8].

There are different types of treatment for breast cancer, depending on its type and the extent of the spread, and patients often receive more than one type of treatment [9]. A treatment plan is a document describing the path for cancer care and can be given to the patient, family or other members of the healthcare team, thereby making everyone aware of the path and the responsibilities when delivering healthcare services [10]. In this regard, it is valuable to use a multidisciplinary team (MDT) approach for optimal treatment management of patients with breast cancer. An MDT meeting for cancer management is known as a tumor board (TB) conference. Applying TB meetings helps determine the best course of treatment for patients and contributes to the provision of standard care services in the field of cancer [11–14]. Several studies have shown the effectiveness of using such an approach in the management of the cancer treatment process [14–16]. However, MDT services are not often available in low-income countries and remote areas, in which shortcomings will reduce patients' quality of life [17].

The healthcare industry around the world has been influenced both directly and indirectly by innovative developments in information and communication technologies (ICTs) [18,19]. One of these influences is the use of ICTs to implement telemedicine systems, which have the potential to increase access to medical services, thereby reducing inequality in the delivery of healthcare services to remote areas [20]. In general, telemedicine provides great opportunities in the areas of patient care, research, education, diagnosis, health data transmission, counseling and training of healthcare professionals, especially for poor and developing countries [21]. The use of telemedicine in oncology is known as teleoncology, and it can be used as a supportive or alternative approach in the screening, diagnosis and treatment of cancer [22]. It has been employed by many healthcare centers worldwide to provide cancer care services for patients in rural and remote areas [23]. Furthermore, the possibility of applying the MDT approach to improve the process of patients' treatment management and follow-up after presenting the treatment plan to them is provided by the use of telemedicine technologies [24]. Therefore, telemedicine is one of the most effective solutions to the problem of providing cancer care services to patients residing in remote areas who do not have access to these services [25]. Although communication platforms such as email, mail, fax and voice or video calls with a colleague are common means of receiving free consultation, the use of a more formal platform such as a telemedicine system as a practical tool is on the rise due to its efficacy and quality and better and more relevant facilities in this context [26]. Improving access to physicians; reducing travel time, cost and stress; speeding up medical processes; and providing more efficient services to patients, as well as offering educational opportunities to healthcare providers, are some of the benefits of using teleoncology systems in the cancer care process [27].

Depending on the type of communication and interaction established between healthcare providers and recipients using telemedicine projects, the development modalities of such projects are divided into three main categories:

1. Store-and-forward (asynchronous interaction), such as using text messaging, chat services and emails;
2. Real-time or videoconferencing (synchronous interaction), such as using free videoconferencing software such as Google Hangouts, Skype and Zoom;
3. Hybrid (a combination of real-time and store-and-forward modalities) [28,29].

The hybrid telemedicine model is the process of providing combined health services (face-to-face and virtual), including online appointment scheduling to provide a better intervention, establish effective online communication between doctors and patients, build trust and so on, yet, when physical examinations and physician presence are needed, face-to-face appointments are arranged [30]. In the hybrid method, prior to establishing synchronous interaction between physicians and patients or other healthcare providers, patient information, including clinical information, medical images and laboratory results, should be provided to the team to review the information through the store-and-forward method [31].

Due to the increasingly high incidence of breast cancer in Iran, it is predicted that the demand for appropriate and timely therapeutic interventions, which should be provided through specialized MDTs, will increase in the coming years. It seems that innovative, technology-based interventions such as telemedicine systems will be needed to address the growing demands for and some shortcomings in the provision of breast cancer treatment services. Furthermore, due to the prevalence of COVID-19 disease in Iran, the need to implement a hybrid teleoncology system is felt strongly, and it will be possible to benefit from the potential capabilities of such a system during the COVID-19 pandemic. Therefore, the present study was conducted for the first time in Iran in line with the existing needs to develop a hybrid teleoncology system and to evaluate its usability with the aim of providing treatment plans for breast cancer patients. In this study, the primary purpose of developing a teleoncology system was to facilitate the establishment of TB meetings and communications between members of MDTs that would result in providing treatment plans for cancer patients, and it was not designed for ongoing patient consultation.

## Materials & methods

This applied developmental research was conducted in four stages. The first stage began with the proposal of an information technology (IT)-based solution to the problem of providing breast cancer patients with treatment plans, especially during the COVID-19 pandemic, through telemedicine. The initial proposal was prepared by comprehensively reviewing research studies addressing telemedicine for breast cancer. The proposal was presented to a committee consisting of experts in the field of telemedicine and clinical specialists with experience in breast cancer treatment. In this step, the experts approved the proposal and its objectives, as well as the method of providing services by the newly proposed intervention and the possibility of evaluating various aspects of it. In the second stage, needs analysis and identification of the requirements of the proposed IT-based intervention were performed. In this regard, the required data elements that were necessary to provide a treatment plan for breast cancer patients were identified. Furthermore, the functional and non-functional requirements of a teleoncology system were determined through interviews with the end-users of the system [32].

In the third stage, a web-based prototype of the system was designed and made available to users when the acceptability and appropriateness of the proposed new solution, based on the results of the first stage, were confirmed and the requirements of the new system, according to the actions of the second stage, were identified. In addition, at this stage of the study, the question-asking protocol was used for identifying deficiencies and drawbacks of the prototype from the users' viewpoint. In this regard, the authors compiled a list of key tasks according to the different roles of the system users. For example, the residents were responsible for recording patients and breast cancer specialists' data, and then the users were asked to perform the assigned tasks. The results of each task were determined by three options: ‘successful’, ‘unsuccessful’ and ‘erroneous’. In order to better understand the deficiencies of the prototype and the users' perspectives, the authors were present as observers and tried to guide the users in performing specific tasks or functions of the system through directional questions during the question-asking protocol.

In the fourth stage, after applying the comments and suggestions provided by users in the previous stage, the MySQL (Structured Query Language) database management system, the Hypertext Preprocessor (PHP) programming language, the HTML and Cascading Style Sheets (CSS) technologies were used to design the final version of the web-based teleoncology software. As previously mentioned, the teleoncology system was developed through a hybrid method in this study. Therefore, Skype software was used to establish synchronous communication between specialists. However, specialists can use the asynchronous method by employing the embedded text messaging and online chat services in the system to exchange views and receive comments and decisions from colleagues. The final version of the teleoncology system was piloted between 25 May and 25 November 2020 to assess its usability and to ensure that the new intervention meets users' expectations. The usability of the system was evaluated with the Persian version of the standard Questionnaire for User Interaction Satisfaction (QUIS). This tool consisted of 32 questions in six sections, including overall reactions to the software (six questions), screening (four questions), terminology and system information (six questions), learning (six questions), system capabilities (five questions) and usability and user interface (five questions). For each question in each section, an answer with a score of 0 (minimum)–9 (maximum) was considered on the basis of a 10-point Likert scale. Finally, the average score was calculated for each part of the questionnaire.

## Results

As mentioned earlier, the present study was conducted to develop a hybrid teleoncology system to provide treatment plans for breast cancer patients. In this regard, the results of the different stages of the research are presented respectively. The results of the first stage, which included the required data elements and technical requirements of the teleoncology system, were presented in an article entitled *“*Required data elements and requirements of a teleoncology system to provide treatment plans for patients with breast cancer*”* [32]. According to studies, in the question-asking protocol, the presence of five users, including the researcher as an expert observer and four end-users, was found to be sufficient for the evaluation [33].

In the second stage, a total of six specialists in the field of breast cancer, including two residents of radiation oncology, two radiation oncologists, one pathologist and one cancer surgeon, were selected by the nonrandom snowball sampling method to participate in the question-asking protocol. The list of tasks assigned to the participants is presented in Tables 1–3 according to their roles in the system and the results of the performance. The results analysis showed that among the key tasks assigned to the participants, tasks such as “enter the data of the clinical tests results”, "edit the content of patient record”, “enter the clinical images" and “print patient record” could not be completed properly by users due to errors in the designed prototype.

**Table 1.** List of key tasks assigned to the residents of radiation oncology.

Row	Task	Participant 1			Participant 2		
		Successful	Unsuccessful	Erroneous	Successful	Unsuccessful	Erroneous
1	Enter to the site via the URL	✓			✓		
2	Create your account/profile in the site	✓			✓		
3	Log in to your account/profile page	✓			✓		
4	Edit your profile and save the changes	✓			✓		
5	Create a new file for the patient record	✓			✓		
6	Enter patient demographic and anthropometric data	✓			✓		
7	Enter patient clinical data	✓			✓		
8	Enter data on the results of a breast physical examination	✓			✓		
9	Enter data on the results of lymph node examination	✓			✓		
10	Enter data on the results of the distant metastasis examination	✓			✓		
11	Enter data on the results of the breast examination (patients who had a previous lumpectomy)	✓			✓		
12	Enter data on the results of the chest examination (patients who had a previous mastectomy)	✓			✓		
13	Enter data on the results of pathology of breast biopsy	✓			✓		
14	Enter data on the results of pathology of lymph node biopsy	✓			✓		
15	Enter data on the results of surgical pathology	✓			✓		
16	Enter the IHC & FISH (or CISH) results of lymph node tissue	✓			✓		
17	Enter the IHC & FISH (or CISH) results of breast mass lesions	✓			✓		
18	Enter the IHC & FISH (or CISH) results of surgical procedures	✓			✓		
19	Enter data on the results of recurrence pathology	✓			✓		
20	Enter the IHC & FISH (or CISH) results of recurrence	✓			✓		
21	Enter data on the pathology results of the metastatic biopsy	✓			✓		
22	Enter the IHC & FISH (or CISH) results of the metastatic tumors	✓			✓		
23	Enter the data on the results of the TNM-based classification system	✓			✓		
24	Enter data on the assessment results of the PDL-1 status in triple-negative patients	✓			✓		
25	Enter the data of the clinical tests results		✓			✓	
26	Enter the clinical images			✓			✓
27	Enter data on the results of the counseling and patient preferences	✓			✓		
28	Save the patient record	✓			✓		
29	Edit the content of patient record			✓			✓
30	Send the completed patient record to the team of physicians providing the treatment plan	✓			✓		
31	Observe the responses by the team of physicians providing the treatment plan	✓			✓		
32	Send the treatment plan provided by the team of physicians to the patient	✓			✓		
33	Search in the records of patients	✓			✓		
34	Print the patient record			✓			✓
35	Log out from your account/profile	✓			✓		
36	Recover your password	✓			✓		
37	Exit from the site	✓			✓		

CISH: Chromogenic in situ hybridization; FISH: Fluorescence *in situ* hybridization; IHC: Immunohistochemistry.

**Table 2.** List of key tasks assigned to the radiation oncologists.

Row	Task	Participant 1			Participant 2		
		Successful	Unsuccessful	Erroneous	Successful	Unsuccessful	Erroneous
1	Enter to the site via the URL	✓			✓		
2	Create your account/profile in the site	✓			✓		
3	Log in to your account/profile page	✓			✓		
4	Edit your profile and save the changes	✓			✓		
5	Observe the list of patient records	✓			✓		
6	Observe the record of a specific patient to propose a treatment plan	✓			✓		
7	Examine the comments and suggestions provided by other specialists in the treatment team	✓			✓		
8	Provide the final treatment plan according to the patient's record and the opinions of other specialists	✓			✓		
9	Send the final treatment plan to the residents of radiation oncology	✓			✓		
10	Print the patient record			✓			✓
11	Search in the records of patients	✓			✓		
12	Log out from your account/profile	✓			✓		
13	Recover your password	✓			✓		
14	Exit from the site	✓			✓		

**Table 3.** List of key tasks assigned to the pathologist and cancer surgeon.

Row	Task	Pathologist			Cancer surgeon		
		Successful	Unsuccessful	Erroneous	Successful	Unsuccessful	Erroneous
1	Enter to the site via the URL	✓			✓		
2	Create your account/profile in the site	✓			✓		
3	Log in to your account/profile page	✓			✓		
4	Edit your profile and save the changes	✓			✓		
5	Observe the list of patient records	✓			✓		
6	Observe the record of a specific patient to propose specialized comments and suggestions according to the patient's record	✓			✓		
7	Provide your specialized comments and suggestions according to the patient's record	✓			✓		
8	Send your comments and suggestions to the residents of radiation oncology	✓			✓		
9	Print the patient record			✓			✓
10	Search in the records of patients	✓			✓		
11	Log out from your account/profile	✓			✓		
12	Recover your password	✓			✓		
13	Exit from the site	✓			✓		

As mentioned earlier, in the question-asking protocol, while the users were using the prototype, the researchers were present as observers and documented the comments, suggestions and modification requests provided by the users. Thereafter, the modifications requested by the users were applied to correct the typos on system term, to change the arrangement of data in different tabs and to add and modify data fields in some tabs. Subsequently, the final version of the web-based teleoncology system was made available to the users (www.teletreatplan.ir) after making corrections and eliminating the shortcomings and deficiencies of the system prototype. It should be noted that the hospitals and medical centers having access to real-time communications tools (internet with suitable bandwidth, computer, camera, microphone and speaker) could use both store-and-forward and real-time methods; otherwise, only the store-and-forward method was used. In the real-time (synchronous) method, Skype software was used to establish videoconferencing between specialists. However, if it was not possible to hold videoconferencing sessions at the same time, or there was no need to use videoconferencing based on the patient's clinical data in the system (there were no complicated conditions for making a decision regarding a treatment plan), communication between specialists was established asynchronously by using text messaging and online chat services.

**Figure 1.**
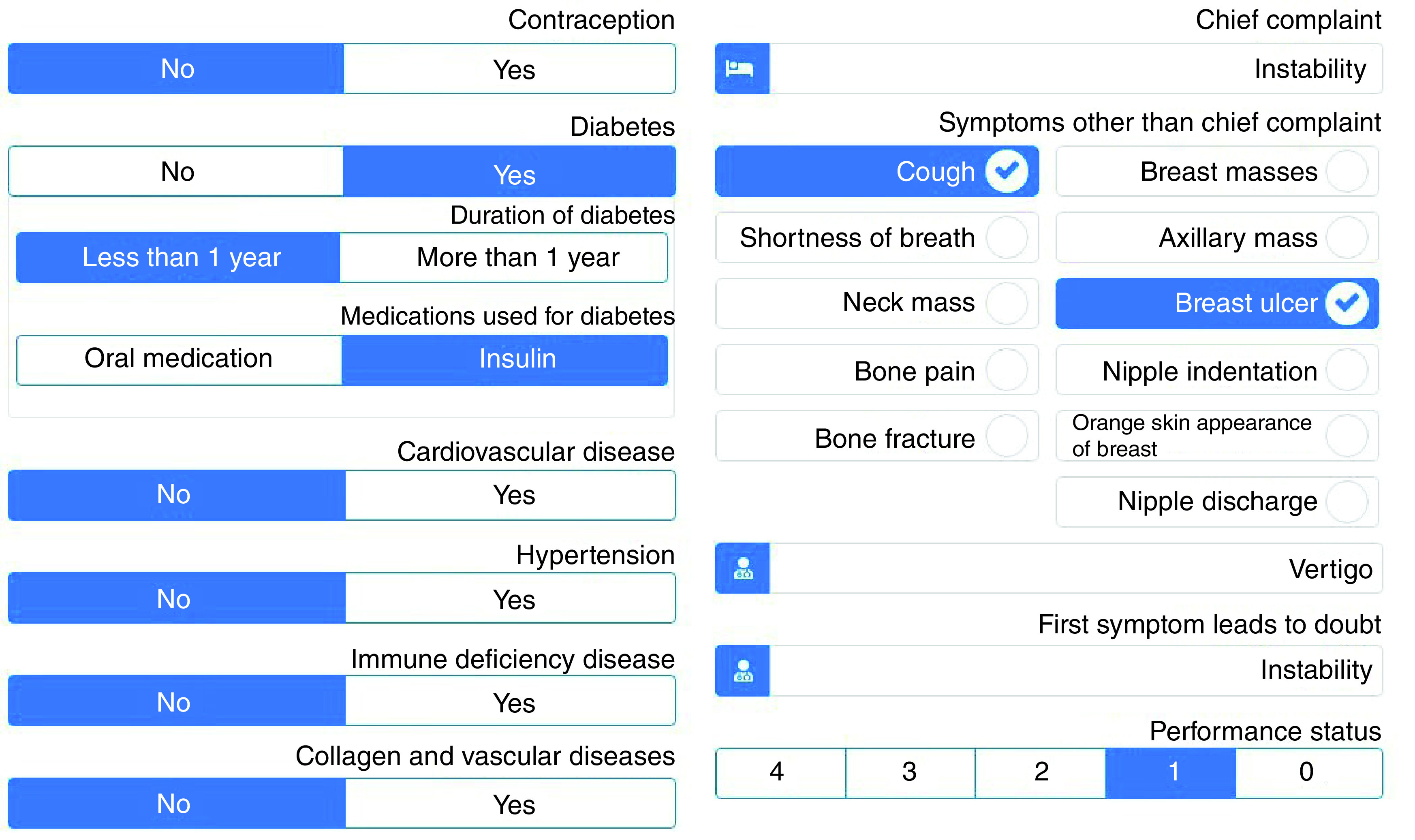
The information/data entered by the residents.

**Figure 2.**
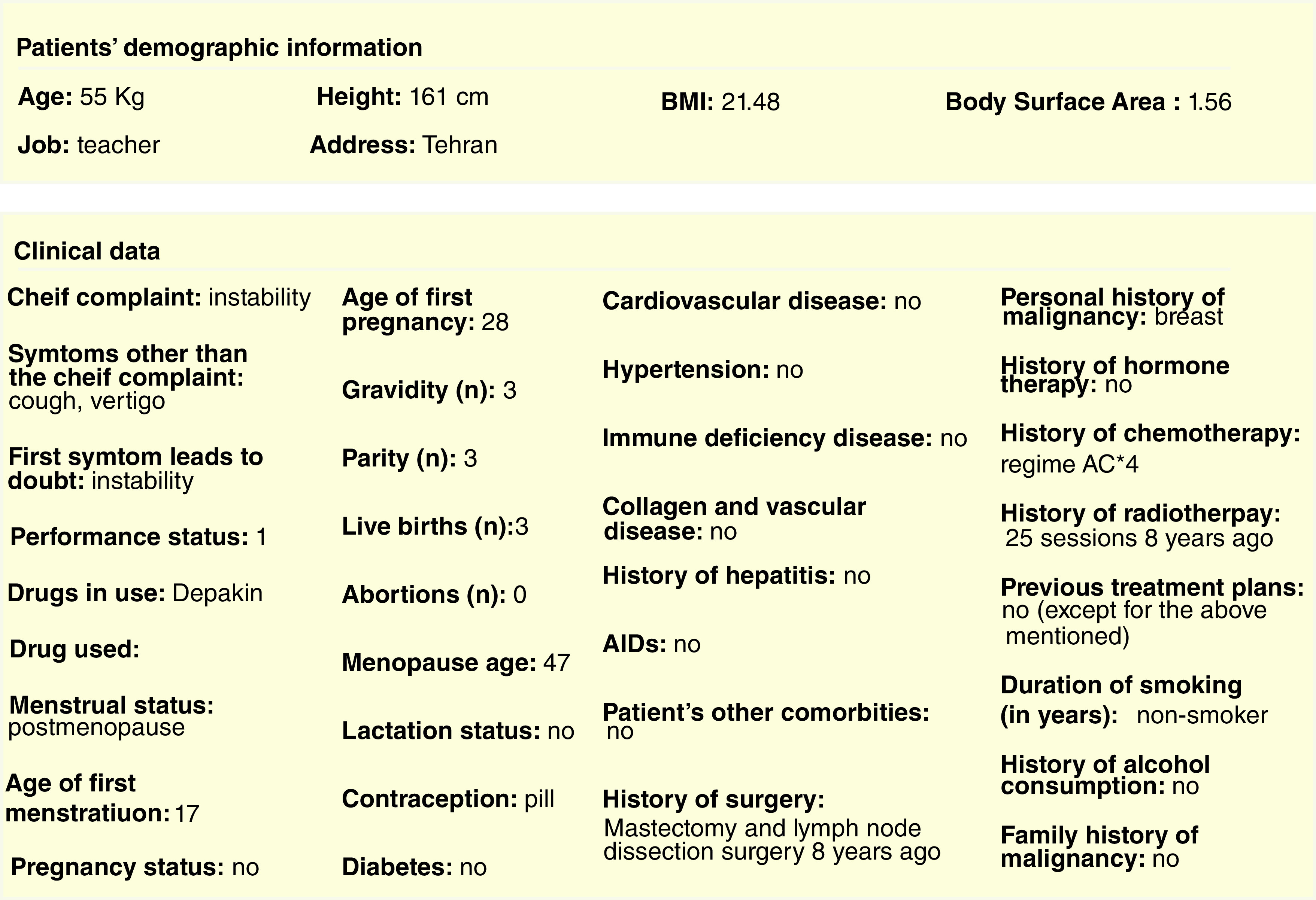
The information/data received by the specialists.

Users who want to use the teleoncology system must complete the initial registration by entering the requested information (first name, last name, national code, medical council code, email address, username, password and role of the user in the system) into the registration form. Since the users of the teleoncology system have different roles and perform certain activities in the system according to their roles and expertise, user roles (including resident of radiation oncology, radiation oncologist, pathologist, radiologist, cancer surgeon, plastic surgeon and patient) are defined in the registration form. After completing the initial registration process, users need to wait for authentication by the system administrator to activate their account in order to access the system through their username and password. For patients to have access to their account and medical records, their records must already be registered by residents in the system. Then, usernames and passwords are provided to the patients to complete their accounts and gain access to their medical records. Therefore, patients can be informed about the treatment plans and programs developed for them by specialists. If a patient's presence in MDT meetings is considered necessary, the patient will be notified through text messaging embedded in the system. There is no doubt that informed consent forms should be obtained before offering the service to patients. Thus, the forms are included in the telemedicine system, and patients should fill them out prior to being offered the service. It should be noted that if the patients and treatment team are willing and satisfied, this new approach will be used to continue the patient treatment process. Figures 1 & 2 illustrate the information entered by the residents and the information received by the specialists, respectively (Supplementary Figures 1 & 2 show the information as presented in the original Arabic of the tele-oncology system).

Upon implementation of the final version of the telecommuting system, the system was available to users for 6 months to test its usability. During this time period, treatment plans for 76 (46%) breast cancer patients referred to the oncology clinic of Shohaday-e-Haft-e-Tir Hospital were presented through a teleoncology system. In addition, 43 videoconferences were held via Skype. The usability of the system was evaluated with the Persian version of the standard QUIS after this 6-month period. The type of sampling at this stage was the non-random judgmental sampling method. The research sample included five patients and 25 specialists (blood and adult cancer specialists, cancer surgeons, radiation oncologists, residents of radiation oncology, pathologists and radiologists). The demographic information of the participants in this stage is presented in Table 4.

**Table 4.** Demographic information of the participants.

Characteristic	Specialists n (%)	Patients n (%)
**Gender**		
Male	12 (40%)	0 (0%)
Female	13 (43.33%)	5 (16.67%)
**Age (years)**		
<31	2 (6.67%)	0 (0%)
31–35	4 (13.34%)	1 (3.33%)
36–40	2 (6.67%)	2 (6.67%)
41–45	7 (23.33%)	1 (3.33%)
>45	10 (33.33%)	1 (3.33%)
**Work experience (years)**		
<5	7 (23.33%)	–
5–10	5 (16.67%)	–
>10	13 (43.33%)	–
**Level of education**		
Clinical specialization	25 (83.34%)	–
Lower than bachelor	–	1 (3.33%)
Bachelor	–	3 (10%)
Above bachelor	–	1 (3.33%)
**Field of study/clinical field**	6 (20%)	–
Radiation oncology		
Residents of radiation oncology	6 (20%)	–
Blood and adult cancer specialists	3 (10%)	–
Pathology	4 (13.34%)	–
Radiology	3 (10%)	–
Cancer surgeons	3 (10%)	–
Others	–	5 (16.66%)

At this stage, the Persian version of the QUIS (seventh edition) was used to evaluate the usability of the system from the users' point of view.

As Table 5 shows, the average scores for each part of the questionnaire from the users' point of view are between 6.1 and 9, respectively. Therefore, it can be concluded that users have generally evaluated different parts of the teleoncology system as ‘good’.

**Table 5.** Results of teleoncology system usability evaluation.

Measures	Mean	Standard deviation
Overall reactions to the software	8.1	0.94
Screen	8.8	0.42
Terminology and system information	7.6	1.08
Learning	8.2	0.91
System capabilities	7.6	1.20
Usability and user interface	8.1	1.01

## Discussion

The worldwide pandemic (COVID-19) has affected many industries, especially the healthcare industry, which requires the use of new methods and technologies to perform interventions such as screening, diagnostic, therapeutic, patient care and patient–physician interactions. Hence, telemedicine is a practical and useful tool improving the provision of various medical services and interventions during the COVID-19 pandemic by using ICT. The results of a study by Yildiz *et al.* on the implementation of a teleoncology system in the field of breast cancer revealed that telemedicine technology is a suitable tool that can be used for providing medical oncology services during the COVID-19 pandemic [34]. Therefore, in this study, the design and development steps of a hybrid teleoncology system aiming to provide treatment plans for breast cancer patients are presented.

The prototype is a very simple model of software programs provided to customers. Ultimately, the final and complete version of the software will be developed and released by applying changes based on customer needs and requests during the software development process [35]. Therefore, the proposed prototype should be examined and evaluated by software users. Since there are different goals for evaluating software systems, various methodological approaches differing in terms of the type of technique and structure are also required [36,37]. The findings of a systematic mapping study, investigating the Usability Evaluation Methods (UEMs) used for web-based software, showed that ‘user testing’ was the most commonly used method among the UEMs. Moreover, the think-aloud method and the question-asking method are two common user testing methods, often employed in the relevant studies [38]. The question-asking method goes one step further than the think-aloud method, in a way that the testers ask questions about the product from the users. This method is efficacious because it creates a better understanding of the user's mental model, and it is possible for the user to interact more with the product while focusing on specific areas. In addition, this method often leads to users' improved performance [39]. Therefore, in the present study, the prototype of teleoncology system was evaluated by using the question-asking method to identify its deficiencies and drawbacks from the user's standpoint. The findings of some studies showed that the question-asking method is superior to other methods for evaluating computer software and identifying deficiencies according to the type of users and the conditions of the study [40–42]. However, studies on computer software evaluation have shown that some of them used the think-aloud method [43–45]. Furthermore, in some studies, a combination of a number of methods has been used to evaluate a prototype of telemedicine systems [46,47].

Teleoncology involves the use of electronic tools and an IT infrastructure to transmit textual data, clinical images and pathology results to assist in clinical diagnosis, counseling, interpretation of pathology results, treatment planning, supportive care and follow-up services for cancer patients [48,49]. A common model for teleoncology projects is the use of real-time videoconferencing, which allows a multidisciplinary oncology team to provide expert advice and feedback to medical centers located in remote and rural areas [50]. This method has been used in most teleoncology projects [25,51–53]. However, in the present study, the teleoncology project was implemented using a hybrid method to propose treatment plans to patients with breast cancer. The reason for using the hybrid method was that in this method the features and capabilities of both the store-and-forward and the real-time methods are combined and used. As a result, medical centers and patients with access to the internet and videoconferencing facilities can use and benefit from the features of the real-time method. The store-and-forward method can be used through easily accessible web browser software by those lacking access to videoconferencing facilities. It is noteworthy that due to the fact that today's laptops and smartphones are equipped with the internet, a web-camera, microphone, speaker and screen, it seems unlikely to face barriers to access and use the teleoncology system developed in this study for patients or specialists.

Connection to Hospital Information Systems (HIS) is based on the rules and policies of the corresponding health institutions and must pass through excessively complicated administrative procedures. Furthermore, since this teleoncology system has been developed as a pilot project, it was not connected to other information systems. Therefore, connection to HIS was not included in the aims and scope of this pilot project, and records of meetings were manually entered into the patients' records. Moreover, due to the fact that patients may incorrectly record specialized information about the disease in their medical records, they were only allowed to change and record their demographic information, and other medical information was recorded in the patients' medical records by the residents. However, the results of some studies have shown that the connection and integration of telemedicine systems with other clinical information systems such as HIS facilitate the process of entering patients' clinical information, improves interoperability between information systems, increases the quality of input data and saves data entry time [54,55]. Therefore, it is recommended that in the early stages of the development process of telemedicine systems the necessary actions be taken to obtain the approval of health centers and the required permissions to connect and integrate with HIS. As mentioned in the previous sections, the usability of the final version of the teleoncology system was evaluated by using the QUIS questionnaire. Similarly, some researchers in their studies have used the QUIS questionnaire to evaluate the usability of telemedicine systems [43,56,57]. In addition, some studies have used the System Usability Scale (SUS) questionnaire to assess usability [46,58]. In general, it seems that various questionnaires have been used in studies where usability assessment was performed with a questionnaire. However, in similar studies in the field of teleoncology, evaluations have focused solely on the final clinical outcomes, and the results of the implementation of telemedicine projects have been compared with the traditional methods. In this regard, a number of consultations and treatment plans provided by the teleoncology projects were compared with the traditional model, and the benefits of these telemedicine programs, including reducing costs and saving time to access health services, have been highlighted [24,25,59–61]. It is noteworthy that in some similar studies in the field of teleoncology, user satisfaction with implemented projects was examined with qualitative [62] or mixed methods (quantitative and qualitative) [63] by using tools such as questionnaires [23,64,65] or interviews [66]; however, the usability of the implemented projects was not evaluated from the users' point of view.

## Conclusion

The hybrid teleoncology system is a suitable and practical alternative to traditional methods of providing treatment plans to breast cancer patients, especially during the COVID-19 pandemic. However, the successful design and development, implementation, acceptance and use of such systems require addressing the end-users in all phases of the system development life cycle, along with receiving feedback and evaluating their satisfaction with the system. It is recommended that further studies be carried out on the designed system by adding the capabilities and features of smartphone applications. The findings of such studies, along with the results of the present study, could sketch out a more thorough picture of a teleoncology treatment plan of management system.

## Future perspective

In our view, with the spread of the internet and ICTs in developing countries, the use of telemedicine to provide screening, treatment, diagnostic and rehabilitation services will increase widely in the field of oncology. With the emergence of crises such as COVID-19 pandemic, patients and professionals are more inclined to use such innovations.

**Summary points**

- This is one of the first studies in Iran that describes the steps in the development, implementation and evaluation of a hybrid teleoncology system for breast cancer. Providing prototypes of software under development allows the software end-users to evaluate the initial design and ultimately build the final product using their suggestions and feedback.
- The usefulness of the question-asking method lies in a better understanding of the user's mental model is created, and it is possible for the user to interact more with the product while focusing on specific areas.
- Implementing a hybrid telemedicine system has the potential of both the store-and-forward and real-time methods.
- The system has the potential of employing the multidisciplinary team approach to improve the process of patient treatment management by the use of a teleoncology system.
- The use of a teleoncology system can provide a treatment plan for breast cancer patients without the need for face-to-face consultation.
- Teleoncology is a suitable and practical tool for providing treatment plans to breast cancer patients, especially during the COVID-19 pandemic.
- Proper implementation and acceptance of the use of teleoncology systems mainly depend on the involvement of end-users, including various specialists and patients, in all stages of its design and development.
