# Supplementary figures and images for: Development and evaluation of a teleoncology system for breast cancer during the COVID-19 pandemic
Source: Future Oncol. 2022 Feb 7:10.2217/fon-2021-0822. doi: 10.2217/fon-2021-0822 (PMC8842715; doi:10.2217/fon-2021-0822)

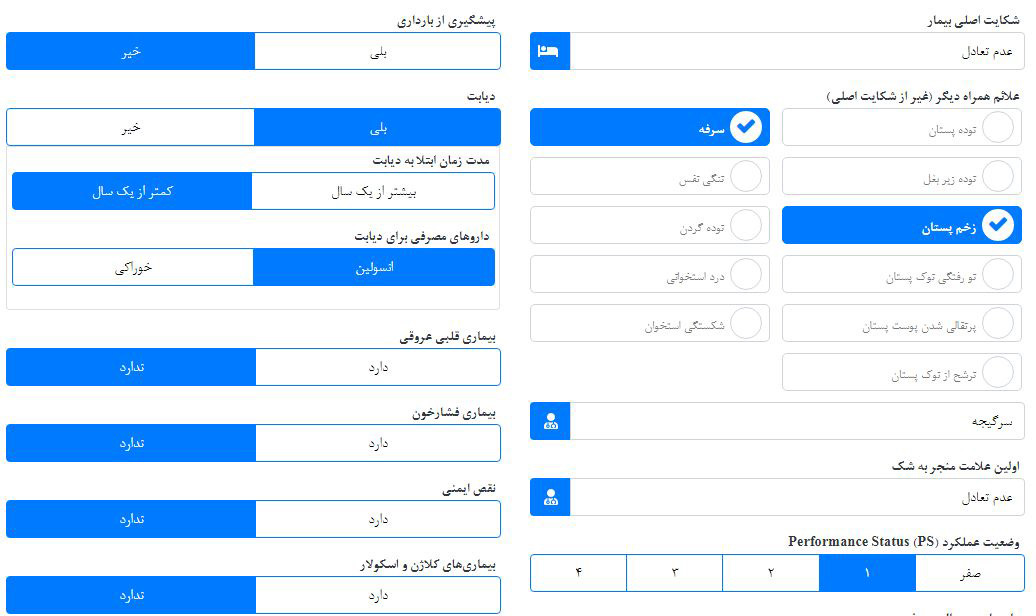

Supplement: Supplementary file 1 [file supplementary-figure-1.jpg]

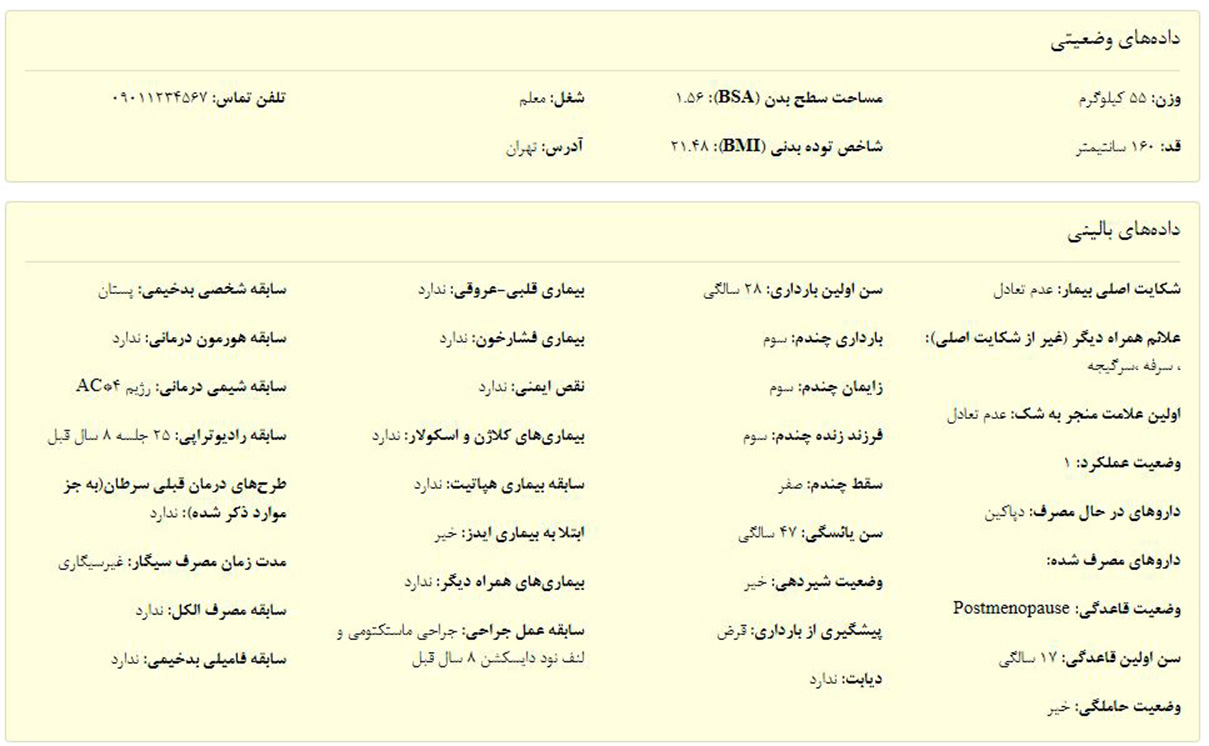

Supplement: Supplementary file 2 [file supplementary-figure-2.jpg]
